# Colonisation with multidrug-resistant organisms among dialysis patients at Universitas Academic Hospital

**DOI:** 10.4102/sajid.v39i1.607

**Published:** 2024-07-09

**Authors:** Godknows Shamhuyashe, Nicoline van Zyl, Cornel van Rooyen, Feziwe Bisiwe, Jolly Musoke

**Affiliations:** 1 Department of Internal Medicine, Faculty of Health Sciences, University of the Free State, Bloemfontein, South Africa; 2 Division of Nephrology, Department of Internal Medicine, Faculty of Health Sciences, University of the Free State, Bloemfontein, South Africa; 3 Department of Biostatistics, Faculty of Health Sciences, University of the Free State, Bloemfontein, South Africa; 4 Department of Medical Microbiology, Universitas Business unit, National Health Laboratory Service, Bloemfontein, South Africa; 5 Department of Medical Microbiology, Faculty of Health Sciences, University of the Free State, Bloemfontein, South Africa

**Keywords:** chronic kidney disease, colonisation, dialysis, multidrug-resistant organisms, Universitas Academic Hospital

## Abstract

**Background:**

While most infections with multidrug-resistant organisms (MDROs) affect colonised people, there is limited evidence on MDRO colonisation in South African dialysis patients.

**Objectives:**

This study evaluated the prevalence of MDRO colonisation among dialysis patients, the resistance patterns of each MDRO and the risk factors for colonisation.

**Method:**

Rectal and nasal swabs were collected from dialysis patients who consented to participate in a 5-month study to identify selected MDROs (April 2021 – August 2021). Specimens were cultured on selected chromogenic media. Data collected included demographics, clinical information from medical records and laboratory results.

**Results:**

Multidrug-resistant organisms were isolated from 17 (23.9%) of the 71 enrolled participants. Of the 23 MDRO strains from rectal swabs (*n* = 71), extended-spectrum beta-lactamase-producing Enterobacterales accounted for 21.1% (15/71), vancomycin-resistant enterococci 2.8% (*n* = 2/71) and carbapenem-resistant Enterobacterales 4.2% (*n* = 3/71). Klebsiella pneumoniae (65.2%, *n* = 15/23) was the most prevalent MDRO. More than 80% resistance to trimethoprim and sulfamethoxazole, cefotaxine, and ciprofloxacin was noted. Significant risk factors included previous hospitalisation, proton pump inhibitor use and antibiotic exposure in the past 6 months.

**Conclusion:**

Multidrug-resistant organisms’ carriage was high in our dialysis population. The infection prevention and control measures need to be revised and strengthened.

**Contribution:**

This study falls within the scope of the SAJID journal as it is the first within sub-Sahara Africa to report that approximately one-fifth of dialysis patients were colonised with MDRO, which is a significant risk for MDRO infections.

## Introduction

The World Health Organization (WHO) has given priority to multidrug-resistant, gram-negative bacteria (MDR-GNB) for control and research because of the pressure they put on healthcare settings.^1^ In patients with chronic kidney disease (CKD), nosocomial infections with multidrug-resistant organisms (MDROs) occur in 2%–5% of cases, leading to higher morbidity and mortality rates.^2^ Patients with CKD on dialysis are more vulnerable to infections because of the azotemic, chronic inflammatory state that alters innate and adaptive immunity and the nature of dialysis devices.^3,4^ After cardiovascular causes, infections are the second leading cause of morbidity and mortality in approximately 50% of dialysis patients.^5^ Colonisation with *Staphylococcus aureus*, particularly methicillin-resistant *Staphylococcus aureus* (MRSA), is associated with a fourfold higher risk of bacteraemia and catheter-related infections in haemodialysis (HD) patients, with vancomycin-resistant enterococci (VRE) having a 2.5-fold risk of bacteraemia.^6,7^ The onset of symptomatic infection usually begins with colonisation. Resistance organisms are more likely to colonise a host. Other species can colonise a host, evolve resistance mechanisms and outwit the host’s defence systems. In Africa, antimicrobial resistance is increasing, according to the WHO’s Global Report on Surveillance.^1^ Of concern are the ‘ESKAPE’ pathogens that include enterococci, *Staphylococcus aureus*, members of the family Enterobacterales, *Acinetobacter* species and *Pseudomonas aeruginosa.*

The epidemiology of colonisation with MDRO varies by region and healthcare setting. Colonisation and infections with MDRO ranging from 3% to over 20% have been reported in patients undergoing dialysis.^8,9,10,11^ Methicillin-resistant *Staphylococcus aureus* colonisation in the dialysis population is estimated to be 6.2%; however, further patient stratification depending on dialysis modality reveals that 7.2% of HD patients are colonised with MRSA, compared to 1.3% of continuous ambulatory peritoneal dialysis (CAPD) patients.^6^ Methicillin-resistant *Staphylococcus aureus* colonisation serves as a reservoir for transmission and is a risk factor for the development of MRSA infection and increased mortality. According to a systematic evaluation of cross-sectional research, 6.2% of dialysis patients have VRE colonisation.^7^ The use of antibiotics such as vancomycin, proximity to other VRE patients, extensive contact with the healthcare system and the presence of multiple comorbid conditions all increase the risk of acquiring VRE in dialysis patients, limiting therapeutic options for serious enterococcal infections.

Few studies of MDR-GNB colonisation rates have been reported in the dialysis population, with one study showing a prevalence of 16%.^12^ Colonisation with carbapenem-resistant Enterobacterales (CRE) has been associated with a 16% overall rate of subsequent infections, most frequently respiratory infections, followed by urinary tract infections, skin and soft tissue infections and primary bloodstream infections.^13^ Prevention of CRE transmission necessitates accurate and rapid laboratory detection methods, which aid in the rapid implementation of infection prevention and control (IPC) measures.^14^ Despite significant physiological differences in the organisms’ colonisation mechanisms such as adherence and biofilm formation, many risk factors for MDRO colonisation are the same. Infections produced by MDRO have a two to five times greater rate of morbidity and mortality than infections brought on by antimicrobial-susceptible organisms, and hence it is imperative to prevent colonisation and infection.

There is little information available on colonisation with MDRO among dialysis patients in South Africa. At the Universitas Academic Hospital (UAH) renal unit, the rate of colonisation with MDRO among dialysis patients is unknown. It is critical to investigate the prevalence of colonisation with resistant microbes and predisposing factors for colonisation.

Vancomycin and amikacin are empirically used as a treatment for patients with suspected catheter-related bloodstream infections and CAPD-associated peritonitis, with further therapy modified according to culture and susceptibility results. These patients may be subjected to a higher risk of MDRO colonisation as a result of the selection pressure created by the administration of these broad-spectrum antibiotics.

This study aimed to determine the prevalence of colonisation and describe the resistance patterns of each MDRO and risk factors for colonisation with MDRO among dialysis patients at the UAH renal unit.

## Research methods and design

### Study design, population and setting

Between 26 April 2021 and 16 August 2021, a prospective descriptive study was conducted at the UAH outpatient HD unit and CAPD clinic, serving about 150 dialysis patients. Universitas Academic Hospital is a 636-bed tertiary-level hospital situated in Bloemfontein, a city in the Free State Province of South Africa. Patients are referred from different peripheral hospitals for assessment for the continuous renal replacement therapy (CRRT) program according to the Department of Health Free State Provincial guidelines.

### Study participants

The following criteria were used for inclusion and exclusion:

Inclusion criteria.

- Patients with end-stage kidney disease (ESKD) dialysing on either HD or CAPD.
- Age 18 years and above at the time of enrolment.

Exclusion criteria:

- Patients on acute dialysis.
- Patients who do not consent to participation in the study.

### Data collection

Participants were interviewed with translations where necessary. Clinical information was collected from MEDITECH electronic patient file system. Laboratory results were printed from the Department of Medical Microbiology. Participants were de-identified using an anonymous study number and an Excel data tool was used to capture each participant’s demographic, clinical and laboratory information. Information collected included the following: demographic information (gender, age and residence); clinical information that entailed the underlying aetiology of the CKD, co-existing conditions, duration of dialysis and its modality, proton pump inhibitors (PPIs) use within the previous 6 months, systemic antibiotic consumption within the previous 6 months and their duration, and duration of admission for the past 6 months before assessment; laboratory results included that the data captured on each study participant data tool were then transferred to an Excel spreadsheet for statistical analysis.

### Specimen collection

Specimens from the anterior nare and rectum were obtained using wet sterile cotton swabs (Amies W/O CH Transystem sterile transport swab; Copan Italia SpA, Brescia, Italy), soaked in 0.9% normal saline. Nasal swabs (inserted about 1 cm into the nostril and circled in both nostrils of a participant) were used to screen for MRSA colonisation. Rectal swabs (collected by inserting a sterile swab approximately 2.5 cm into the anal canal and waiting for 10 s) were screened for VRE, extended-spectrum beta-lactamase (ESBL)-producing Enterobacterales and carbapenem-resistant organisms (CROs). Sample collection was done by the principal investigator. All specimens were immediately sent to the microbiology laboratory for processing within 24–48 h of collection.

### Microbiology methods

**FIGURE 1:**
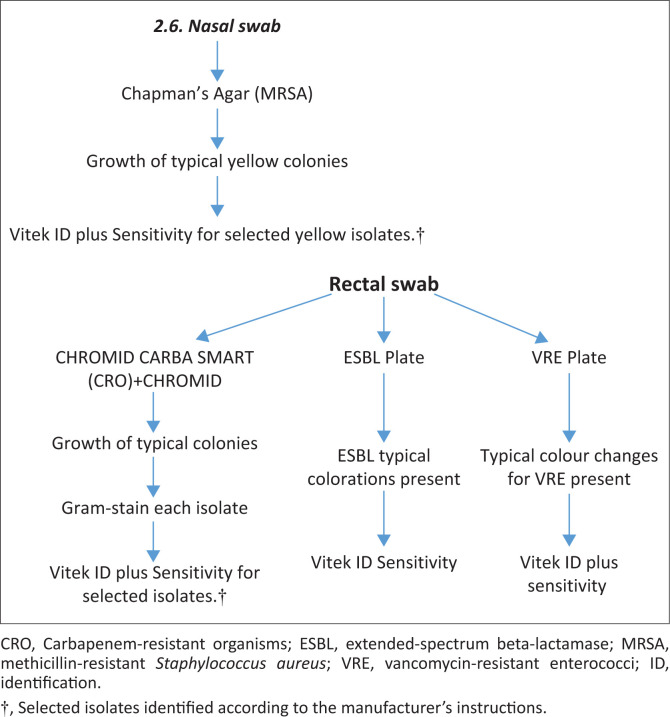
Flow diagram showing microbiological methods.

All specimens were processed at the National Health Laboratory Services (NHLS), Medical Microbiology, Universitas Academic Laboratories, Bloemfontein. The screening of selected MDROs (ESBL, MRSA, VRE and CRE) was conducted as per the NHLS standard operating protocol (SOP) as shown in Figure 1. Clinical specimens were inoculated on commercially available, selective, chromogenic media and incubated at 37°C for 24 h. Suspected colonies were recognised by their characteristic colour changes as per manufacturer’s guidelines. For MRSA identification, the nasal samples were cultured on Chapman’s agar Mannitol salt (NHLS-DMP, South Africa). Colonies morphologically suggestive of *S. aureus* (yellow colonies) were confirmed by Gram staining, catalase test and standard tube coagulase test (coagulase-positive would be suggestive of *S. aureus*). Identification of VRE was done with rectal swabs being inoculated onto chromogenic media, Brilliance VRE agar (Oxoid, UK) plates. Characteristic colonies of VRE grew as either indigo-purple colonies (*Enterococcus faecium*) or light blue colonies (*Enterococcus faecalis*).

Identification of ESBL-producing Enterobacterales was done using chromogenic media, ChromID ESBL Bio-Merieux agar (Bio-Merieux, Marcy I’Etoile, France). ChromID CARBA SMART/OXA-48 Bio-Merieux agar (Bio-Merieux, Marcy I’Etoile, France) was used for the identification of CRE. The CARB component was inoculated first followed by the OXA-48 component using the same rectal swab. CARB-resistant isolates were sent to the reference laboratory for broth microdilution.

Selected bacterial isolates were confirmed for antibiotic susceptibility testing (AST) and species identification using the VITEK-2 system (Bio-Merieux, France) according to the manufacturer’s instructions. The identification and susceptibility card types used were gram-positive (GP) and gram-negative (GN), on the automated VITEK-2 system. Formulations for AST used were Clinical Laboratory Standards Institute (CLSI) compliant. The minimal inhibitory concentration (MIC) of antibiotic susceptibility and interpretation was done using the CLSI 2021 Criteria and definitions.^15^ Isolates were defined as multidrug-resistant if resistant to at least one antibiotic in three or more antimicrobial drug classes that are commonly prescribed. Quality control (QC) procedures were used to assure the accuracy of microbial identification and AST utilising the American Type Culture Collection (ATCC) strains.

All participants were informed about the test results by the principal investigator.

### Ethical considerations

The University of Free State’s Health Sciences Research Ethics Committee (HSREC) granted its approval for the study’s ethical conduct (UFS-HSD2020/1681/2601) before data and specimen collection. Permission was also granted by the Department of Health of the Free State Province and the NHLS. Informed consent was obtained after explaining the purpose of the study to the participants. Data collected were de-identified, processed and analysed on a password-encrypted electronic device with access limited to only the principal investigator and supervisors. Patients who had positive results were informed to improve infection control measures.

### Statistical analysis

Data were coded and entered on an Excel spreadsheet and analysed by the Department of Biostatistics University of Free State using the SAS system for Windows Software. Numerical variables were expressed as mean ± standard deviation, medians (interquartile ranges) and compared using the Wilcoxon-Mann-Whitney test. Categorical data were expressed as percentages using the chi-squared or Fisher exact test where appropriate. The level of statistical significance was set at a *p*-value < 0.05.

## Results

A total of 71 participants consented to both nasal and rectal swabbing. A total of 142 swabs (71 nasal, 71 rectal) were obtained from these participants. Characteristics of participants are shown in Table 1. The study population’s median age was 41.0 years (IQR 20–62). The majority were males 46 (64.8%) and 25 (35.2%) were females. Approximately 66% (*n* = 47) of the study population lives in the township, and 70 (98.6%) had normal mobility with 1 (1.4%) having limited mobility (using a wheelchair). Forty-two participants (59.2%) had hypertensive nephrosclerosis as the underlying kidney disease, 5/71 had human immunodeficiency virus (HIV)-associated nephropathy (7.0%), 6/71 missed glomerulonephritis (8.5%), diabetic nephropathy-1 (1.4%) and 17 (23.9%) had other conditions as the underlying cause of kidney diseases such as lupus nephritis, autosomal-dominant polycystic kidney disease, tubulointerstitial nephritis and uveitis syndrome and immunoglobulin A nephropathy. Forty-seven participants (66.2%) had one or more comorbidities unrelated to the aetiology of CKD such as ischaemic heart diseases, depression and hyperthyroidism. The majority of participants were on CAPD, 49 (69.0%) with 22 (31.0%) being on HD.

**TABLE 1:** Characteristics of participants (*N* = 71).

Characteristics	Variable	Colonised (*n* = 17)†	Noncolonised (*n* = 54)‡	Total (*N* = 71)§		*p*
				*n*	%	
Age (years)	-	-	-	-	-	0.6177
Mobility status	-	-	-	-	-	1.0000
	Normal	17	53	70	98.6	-
	Limited	0	1	1	1.4	-
Gender	-	-	-	-	-	0.5659
	Male	12	34	46	63.0	-
	Female	5	20	25	37.0	-
Primary residence	-	-	-	-	-	1.0000
	City	6	17	23	32.4	-
	Township	11	36	47	66.2	-
	Rural	0	1	1	1.4	-
Underlying chronic kidney disease	-	-	-	-	-	0.0617
	Hypertensive nephrosclerosis	8	34	42	59.2	-
	HIV-associated nephropathy	3	2	5	7.0	-
	Diabetic nephropathy	1	0	1	1.4	-
	Missed glomerulonephritis	3	3	6	8.5	-
	Others	2	15	17	23.9	-
Smoking history	-	-	-	-	-	0.2785
	Smoker	4	7	11	15.5	-
	Ex-smoker	2	16	18	25.4	-
	Non-smoker	11	31	42	59.1	-
Alcohol consumption history	-	-	-	-	-	0.7595
	Yes	0	2	2	2.8	-
	Former	10	25	35	49.3	-
	No	7	27	34	47.9	-
Type of dialysis	-	-	-	-	-	0.6596
	Haemodialysis	6	16	22	31.0	-
	Peritoneal	11	38	49	69.0	-
Dialysis access	-	-	-	-	-	0.6630
	Cuffed vein catheter	5	11	16	22.5	-
	Arteriovenous fistula	1	5	6	8.5	-
	Tenckhoff catheter	11	38	49	69.0	-
Duration on dialysis (years)	-	-	-	-	-	0.3719
	< 3	6	28	34	47.9	-
	3–5	6	11	17	23.9	-
	> 5	5	15	20	28.2	-
Hospitalisation past 6 months	-	-	-	-	-	0.0068
	Yes (< 7 days)	6	13	19	26.8	-
	Yes (> 7 days)	7	8	15	21.1	-
	No	4	33	37	52.1	-
Antibiotic use for the past 6 months	-	-	-	-	-	0.0029
	Yes (< 7 days)	4	8	12	16.9	-
	Yes (> 7 days)	7	8	15	21.1	-
	No	5	39	44	62.0	-
Proton pump inhibitor use past 6 months	-	-	-	-	-	0.0145
	Yes	11	17	28	39.4	-
	No	6	37	43	60.6	-
Nasal swab collected	Yes	-	-	71	100.0	-
Rectal swab collected	Yes	17	54	71	100.0	-

† , Mean age (years) = 40;

‡ , Mean age (years) = 42;

§ , Mean age (years) = 41.

**FIGURE 2:**
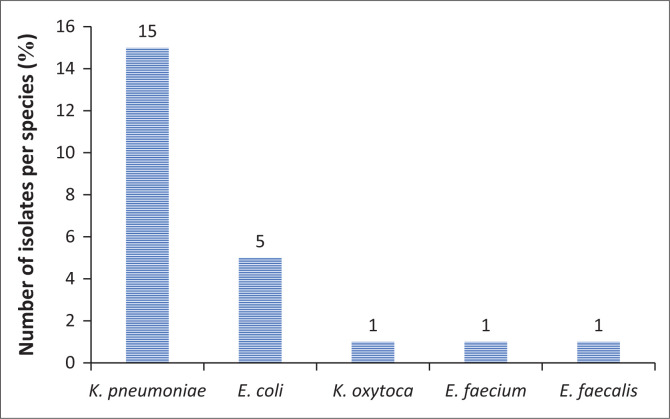
Multidrug-resistant organisms isolated from dialysis patients (*N* = 17) at Universitas Academic Hospital.

Seventeen patients were colonised with MDROs, of which a total of 23 MDROs were isolated from rectal swabs, and no MRSA isolates were detected from nasal swabs. The prevalence of colonisation with MDRO among our study dialysis population (CAPD and HD) was 23.9% (17/71), with males being more frequently colonised than females. In terms of dialysis type, the prevalence rate of colonisation among CAPD patients 9/17 (52.9%) was slightly higher than in HD patients 8/17 (47.1%). Pathogens isolated are shown in Figure 2. These included *Klebsiella pneumoniae* (65.2%, *n* = 15/23), *Escherichia coli* (21.7%, *n* = 5/23), *Klebsiella oxytoca* (4.3%, *n* = 1/23), *E. faecium* (4.3%, *n* = 1/23) and *E. faecalis* (4.3%, *n* = 1/23). In three of the participants, there was mixed growth of *Klebsiella* species and *E. coli*. One participant had a mixed growth of *E. faecium* and *E. coli.* No *S. aureus* isolates were detected during the study period and multivariate analysis was not performed for MRSA carriage. Coagulase-negative staphylococcus (CNS) was detected in the nasal swabs at 81.7% (*n* = 58/71).

Among samples with VRE, 2/71 (2.8%), 1 participant had *E. faecium* with the other one being *E. faecalis* on the VRE plates and both patients were on HD. Both species had high resistance levels of 100% to gentamicin, streptomycin, ciprofloxacin, erythromycin, tetracycline and teicoplanin. The *E. faecium* isolate also demonstrated resistance to benzylpenicillin and ampicillin.

**FIGURE 3:**
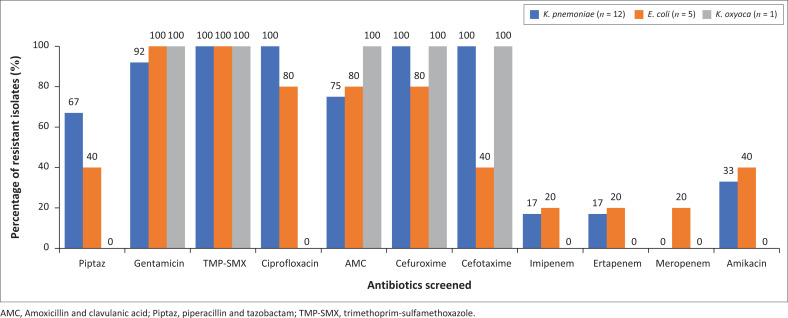
Percentages of extended-spectrum beta-lactamase-producing Enterobacterales resistance patterns (*N* = 17) from the extended-spectrum beta-lactamase plates of dialysis patients at Universitas Academic Hospital.

ESBL-producing Enterobacteriaceae colonization was detected in 15/71 (21.1%) participants from the ESBL plates. This comprised the following organisms: *K. pneumoniae* (12/15 isolates, 80%), *E. coli* (5/15 isolates, 33.3%) and *K. oxytoca* (1/15 isolates, 6.7%). Two participants had a mixed growth of *K. pneumoniae* and *E. coli*, with one participant having a mixed growth of *E. coli* and *K. oxytoca*. The distribution and resistance patterns of these bacteria are shown in Figure 3. *K. pneumoniae* isolates expressed high resistance to ciprofloxacin (12/12 isolates, 100%), trimethoprim and sulfamethoxazole (12/12 isolates, 100%), piperacillin and tazobactam (8/12 isolates, 67%) and gentamicin (11/12 isolates, 92%). The isolate with *K. oxytoca* was resistant to gentamicin (100%), cefotaxime (100%), trimethoprim and sulfamethoxazole (100%) and cefuroxime (100%). *E. coli* isolates showed high resistance to gentamicin (5/5 isolates, 100%), trimethoprim and sulfamethoxazole (5/5 isolates, 100%) and ciprofloxacin (4/5 isolates, 80%). Resistance to amikacin by *K. pneumoniae* and *E. coli* was 33% (4/12 isolates) and 40% (2/5 isolates), respectively. All isolates were 83% susceptible to carbapenems, which included ertapenem and imipenem, with meropenem having 100% susceptibility. Males were colonised more frequently than females with ESBL-producing Enterobacteriaceae.

The CARB-OXA plate identified CRE in 3 of the 71 rectal swabs (4.2%). *K. pneumoniae* was isolated from three male participants on both the CARB and OXA plates. All three isolates were resistant to ertapenem, amoxicillin and clavulanic acid, ampicillin, piperacillin and tazobactam, cefuroxime, cefoxitin, cefotaxime, cefepime and ceftazidime. One isolate was resistant to amikacin. All three participants’ isolates were sensitive to colistin. Two of the participants were admitted in the previous 6 months and received PPIs. Vancomycin and amikacin were prescribed for more than 7 days to all three participants, and no carbapenems were used.

**FIGURE 4:**
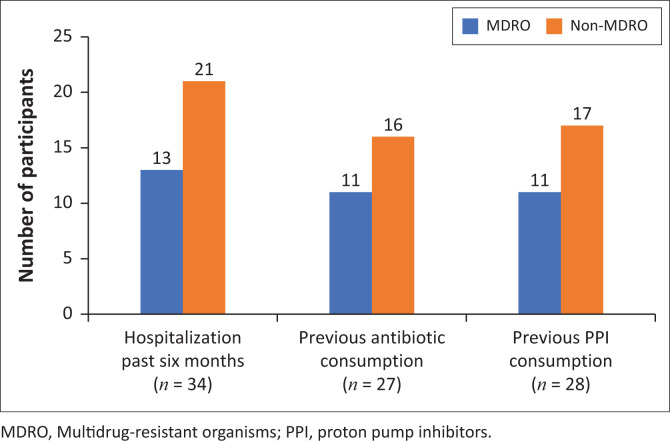
Risk factors associated with multidrug-resistant organisms’ colonisation in the 6 months before study enrolment.

Risk factors analysis for colonisation is shown in Figure 4. Clinical history revealed that 47.9% (*n* = 34/71) of participants had been hospitalised in the last 6 months, of which 38.2% (*n* = 13/34) ended up having MDRO colonisation (*p* = 0.0068). The average hospital stay was 9 days with three participants having been admitted twice before. In the previous 6 months, 38.0% (*n* = 27/71) of the study group were treated with antibiotics, with 40.7% (*n* = 11/27) becoming colonised with MDRO (*p* = 0.0029). Vancomycin and ampicillin were the antibiotics frequently used during treatment. Treatment with PPI (lansoprazole or omeprazole) was given to 39.4% (*n* = 28/71) of the participants in the previous 6 months, with MDRO colonising 39.3% (*n* = 11/28) of the participants (*p* = 0.0145).

## Discussion

In this study, the prevalence of colonisation with MDRO was 23.9% (17/71). This demonstrated that MDRO colonisation is present in approximately one-fifth of our dialysis patients. This is of concern as the literature states that there is a four- to sixfold increased risk that colonisation can lead to infection. Treatment with antibiotics within the previous 6 months for more than 7 days, consumption of PPIs and previous admission were independently associated with an increased risk of colonisation with MDROs. With this, MDRO treatment options are limited and are a clinical and public health concern.

Data on colonisation among dialysis patients with MDROs in South Africa are limited. Several studies in different regions and healthcare settings (mainly developed countries) have reported the prevalence of MDRO colonisation among dialysis patients with frequencies ranging from 3% to over 20%.^6,7,11^ One study at our centre showed that gram-positive organisms accounted for 73% of CAPD-associated peritonitis, of which the most common organism was CNS.^16^ Previously reported carriage rates for MRSA in CAPD patients at our facility were higher, 22.7% as compared to our study with 0%.^17^ In another study in Egypt, the MRSA carriage rate in HD patients was 16.4%, also higher than our findings.^18^ The difference observed could be because of the latter study being conducted over a longer duration. During the study period, no additional research on MRSA infections at our unit was conducted to see whether there had been a decline. In addition, in our study, only one anatomical site was screened. Previous studies have reported that nasal screening has a sensitivity of only 84% in detecting MRSA colonisation. A combination of swabs from the nostrils, perianal and groin has been shown to have the highest rate of MRSA colonisation of more than 90%.^19,20,21^ The risk of infection may not be from patient colonisation but from colonised healthcare workers with subsequent MDRO transmission to patients.^22^

Our prevalence for VRE carriage rate of 2.8% was lower than the prevalence of 6.2% from the previous meta-analysis of various dialysis centres in developed and developing countries.^7^ The lower prevalence of VRE colonisation is reassuring as vancomycin is one of our institution’s empiric antibiotics. Vancomycin-resistant enterococci colonisation can have a regional impact, meaning the presence in one hospital can result in an increase in prevalence to another hospital via patient transfer or the use of common hospital transportation by patients coming for reviews.^23^ Contact precautions should be followed for patients with VRE infection or colonisation.

In our study, ESBL-producing Enterobacteriaceae colonisation (21.1%) was prevalent. The prevalence observed is comparable to other studies where the prevalence ranged from 6.5% to 37%.^12,24^ *K. pneumoniae* was the predominant isolate in this study, 82.4%, followed by *E. coli* (29.4%). This is consistent with previous research in dialysis patients where these microorganisms were prevalent.^12,25^ All CRE carriers (4.2%) were colonised with *K. pneumoniae*. Our findings support previous research that showed that the most prevalent multidrug-resistant pathogens among dialysis patients were gram-negative bacteria.

Vancomycin and amikacin showed low resistance. They are prescribed as first-line treatments at our unit for CAPD sepsis, catheter-related infections and bloodstream infections. Hospital-acquired infections (HAIs) are treated with piperacillin and tazobactam. We had more than 80% resistance to our selected antibiotics of ampicillin, cefotaxime, ciprofloxacin, trimethoprim and sulfamethoxazole. This means that a quarter of patients would be placed on last-resort antibiotics. This increases the chance of treatment failure when third-generation cephalosporins and quinolones are used to treat common infection syndromes, including pneumonia and urinary tract infections. Because of the widespread use of antibiotics, particularly in tertiary institutions, there is increased selection pressure, which is likely to result in changes in resistance patterns. Although our study did not address infections that are caused by MDROs, colonisation is a prerequisite for subsequent multidrug-resistant infections.^6,26,27^

Various studies have analysed MDRO risk factors, comorbidities, previous antibiotic exposure, medical devices and other variables with different outcomes.^21^ With our analysis of risk factors, we observed that previous antibiotic use, PPI use and admission were strong predictors of colonisation with MDRO. Antimicrobial resistance can be reduced through prudent antibiotic use based on local antimicrobial stewardship programs. Eleven of the participants who used PPI ended up being colonised by MDROs (39.3%). In a recent systematic review and meta-analysis, gastric acid suppression was found to be associated with increased odds of intestinal MDROs colonisation by altering gut microbiome composition and increasing the survival of bacteria that pass through the stomach.^28^ Evaluation of risk factors associated with MDRO colonisation is critical in high-risk patient identification and could be useful for implementing effective strategies for the management of MDRO colonisation/infection. Techniques that can be used to reduce colonisation include decolonisation strategies, contact precautions for confirmed cases and surveillance cultures. Therefore, large studies should be carried out to validate these results.

### Limitations

The small sample size and short duration of the study may have contributed to not detecting MRSA in this study and its colonisation being evaluated on a single nasal anatomical site, even though the site swabbed is the main site. Environmental samples, medical devices and healthcare staff were not included in the study. Our study was limited to microbiological surveillance and did not examine interventions to lower colonisation rates or nosocomial infections brought on by MDRO. Furthermore, other MDROs like nonfermenters were not evaluated and colonisation results were not compared with HAI in the unit. Molecular methods could have detected more MDROs in our study. Future studies may consider genotyping of isolates.

## Conclusion

In summary, the study showed that 23.9% of CAPD and HD patients were colonised with MDRO. A high resistance to certain selected antibiotics was shown with a low resistance level to our empirical antibiotics which is reassuring. Patients who have been previously admitted and used antibiotics are at high risk of MDRO colonisation. The implication is that local antibiotic stewardship, as well as healthcare worker hygiene and barrier nursing, should be strengthened. There is a need for high-level alertness and an interdisciplinary approach including infectious disease specialists to address the colonisation with MDRO among dialysis patients. Future research should aim at studying the clinical implications of MDRO colonisation, decolonisation strategies and the involvement of healthcare workers and the environment as risk factors for MDRO colonisation in dialysis patients at our facility.
